# Patterns of psychopathology and cognition in sex chromosome aneuploidy

**DOI:** 10.1186/s11689-021-09407-9

**Published:** 2021-12-15

**Authors:** Srishti Rau, Ethan T. Whitman, Kimberly Schauder, Nikhita Gogate, Nancy Raitano Lee, Lauren Kenworthy, Armin Raznahan

**Affiliations:** 1 Center for Autism Spectrum Disorders and Division of Neuropsychology, Children’s National Health System, Washington DC, USA; 2 Section on Developmental Neurogenomics, Human Genetics Branch, National Institute of Mental Health, Bethesda, MD USA; 3 The Department of Biochemistry & Molecular Medicine, The George Washington University Medical Center, Washington DC, USA; 4 Department of Psychology, Drexel University, Philadelphia, PA USA

**Keywords:** Sex chromosome aneuploidy, Sex chromosomes, Psychopathology, Cognition, Neurogenetic conditions

## Abstract

**Background:**

Sex chromosome aneuploidies (SCAs) are a collectively common family of genetic disorders that increase the risk for neuropsychiatric and cognitive impairment. Beyond being important medical disorders in their own right, SCAs also offer a unique naturally occurring model for studying X- and Y-chromosome influences on the human brain. However, it remains unclear if (i) different SCAs are associated with different profiles of psychopathology and (ii) the notable interindividual variation in psychopathology is related to co-occurring variation in cognitive ability.

**Methods:**

We examined scores for 11 dimensions of psychopathology [Child/Adult Behavior Checklist (CBCL)] and general cognitive ability [full-scale IQ (FSIQ) from Wechsler tests] in 110 youth with varying SCAs (XXY = 41, XYY = 22, XXX = 27, XXYY = 20) and 131 typically developing controls (XX = 59, XY = 72).

**Results:**

All SCAs were associated with elevated CBCL scores across several dimensions of psychopathology (two-sample *t* tests comparing the euploidic and aneuploidic groups [all |T| > 9, and *p* < 0.001]). Social and attentional functioning were particularly sensitive to the carriage of a supernumerary Y-chromosome. In particular, the XYY group evidenced significantly more social problems than both extra-X groups (Cohen’s *d* effect size > 0.5, Bonferroni corrected *p* < .05). There was marked variability in CBCL scores within each SCA group, which generally correlated negatively with IQ, but most strongly so for social and attentional difficulties (standardized *β*, − 0.3). These correlations showed subtle differences as a function of the SCA group and CBCL scale.

**Conclusions:**

There is domain-specific variation in psychopathology across SCA groups and domain-specific correlation between psychopathology and IQ within SCAs. These findings (i) help to tailor clinical assessment of this common and impactful family of genetic disorders and (ii) suggest that dosage abnormalities of X- and Y-linked genes impart somewhat distinct profiles of neuropsychiatric risk.

**Supplementary Information:**

The online version contains supplementary material available at 10.1186/s11689-021-09407-9.

## Introduction

Sex chromosome aneuploidies (SCA) are a collectively common family of neurogenetic disorders that arise due to carriage of an atypical number of X- and/or Y-chromosomes other than the typical female (XX) or male (XY) complement [1]. This family of disorders encompasses several distinct karyotype groups including XXY (Klinefelter), XYY, XXX (Trisomy X), and XXYY syndrome, among others. As detailed below, these conditions are broadly characterized by increased rates of psychopathology and cognitive impairment [2]. Here, we use the term psychopathology to refer to a range of social-emotional and behavioral difficulties reported in SCA that may or may not reach clinically elevated/ diagnostic thresholds. However, to date, studies of psychopathology have tended to focus on one karyotype group or one domain of psychiatric symptomatology at a time. Therefore, few studies have made direct comparisons of different domains of psychopathology across different SCA groups, or tested if the SCA group modifies relationships between co-occurring variation in psychopathology and cognitive ability. Addressing these gaps in knowledge would help to clarify genotype-phenotype relationships within SCAs, which could in turn inform both clinical and neuroscientific understanding of these disorders.

There is convergent evidence across several studies for greater psychopathology in SCAs as compared to typically developing euploidic controls [1–3]. Conditions with an extra X-chromosome (e.g., Klinefelter’s syndrome [47, XXY; KS]; Trisomy X [47, XXX]) have been associated with increased rates of ADHD (with inattention rather than hyperactivity/impulsivity causing greater impairment), executive dysfunction, anxiety, depression, and social difficulties [1, 3–7]. Conditions with an extra Y-chromosome (e.g., 47, XYY) have been associated with increased problems with hyperactivity, defiance, conduct problems, social problems, and low frustration tolerance [8–10], with diagnoses of ADHD and conduct problems being most common [11, 12]. Sex chromosome tetrasomies (e.g., XXYY, XXXY) are substantially rarer than trisomies and appear to be associated with more pronounced behavioral and socio-emotional difficulties [13]. The most prevalent domains of psychopathology in XXYY syndrome include impulsivity, anxiety, temper tantrums, and social difficulties [14–18], with diagnoses of ADHD and mood disorders being most common [19]. Thus, research to date has helped to quantify different aspects of psychopathology in individual SCA groups, and individual domains of psychopathology across multiple SCA groups [8, 20]. However, we still lack quantitative comparisons of multiple dimensions of psychopathology across several SCAs [10]. This undertaking is important considering the wide range of symptoms and symptom severity observed among individuals both within and across SCA groups. Such approaches have proved fruitful in other clinical contexts (e.g., the CBCL-Dysregulation Profile [CBCL-DP [21–28];]), which strongly motivates pursuing their application in SCA research.

A second need in SCA research is to better understand the nature of associations between psychopathology and cognitive ability. Both domains are altered by SCA, and both show high interindividual variation across patients. However, we lack a detailed characterization of how these two key clinical outcomes are interrelated. With regard to SCA effects on IQ, there is a recognized inverse relationship between supernumerary sex chromosome count and IQ [29, 30]. Whereas the sex chromosome trisomies (XXY, XYY, and XXX syndromes) are all associated with an ~ 10 point reduction in full-scale IQ *on average* (slightly larger decrements for verbal as compared to performance IQ), reported mean IQs are typically lower in XXYY syndrome [8, 19, 20, 30, 31]. However, there is marked interindividual variability in IQ within all SCA groups [32–34], and it remains unclear how this is related to the co-occurring interindividual variation that has been described for psychopathology. Work in non-SCA groups suggests there can be complex correlations between clinical variation in IQ and psychopathology [35–45], but the few studies addressing this question in SCA have focused on individual domains of psychopathology [8], prompting the clinically important question of whether some psychiatric features are more strongly coupled to cognitive ability than others in SCA.

The current study aimed to begin addressing the open questions above through analysis of previously unpublished data on psychopathology from a multi-karyotype cohort of individuals with SCA. We first sought to directly compare SCA groups to each other across a range of psychopathology domains, hypothesizing that SCA groups would differ in their profiles of psychopathology. We next considered variations in IQ and psychopathology across individuals with SCAs to test if correlations between these two clinical features varied as a function of psychopathological domain, SCA group, or both. We hypothesized that the magnitude of IQ-psychopathology coupling would vary for different domains of psychopathology, as seen in other clinical groups [43], and possibly further as a function of SCA karyotype.

## Methods

### Participants

One hundred and ten individuals of varying SCA karyotypes (XXY = 41, XYY = 22, XXX = 27, XXYY = 20) and 131 typically developing euploidic controls (TD; XX = 59, XY = 72) participated in this study, which was conducted at the National Institute of Mental Health (NIMH) Intramural Research Program (Clinical trial reg no. NCT 00001246, clinicaltrials.gov; NIH Annual Report Number, ZIA MH002794-13; Protocol number: 89-M-0006). Participants with SCA were recruited through SCA support organizations and NIMH websites. Typically developing controls were recruited through the NIH Healthy Volunteer office. Inclusion criteria for the SCA groups were (i) a non-mosaic SCA diagnosis confirmed by karyotype testing, and (ii) no history of brain injury or comorbid neurological disorder. Inclusion criteria for typically developing controls were no history of neurological, neurodevelopmental, or psychiatric illness. Data from typically developing controls were primarily included as a benchmark for observed levels of psychopathology and psychopathology-IQ coupling in SCA groups; substantive group comparisons were restricted to SCA groups. Sample demographics are described in Table 1. This study was approved by the NIH Combined Neuroscience Institutional Review Board. All participants and/or their parents provided informed consent or assent, as appropriate. All study protocols were completed at the NIH Clinical Center in Bethesda, Maryland.

**Table 1:** Sample demographics

								Statistic		
	Total	XX	XY	XXX	XXY	XYY	XXYY	SCA vs. controls	Comparing all groups	Comparing SCA groups only
Total *N*	241	59	72	27	41	22	20			
Age, M (SD) [range 4.9 to 25.8 years]	13.3 (4.3)	13.2 (3.3)	13.2 (3.3)	12.3 (4.6)	14.1 (5.2)	12.3 (4.8)	14.6 (6.5)	*t* = − 0.4	*F* = 1.2	*F* = 1.3
Prenatal diagnosis (*N*)	49 (45%)	NA	NA	20 (74%)	21 (51%)	8 (36%)	0	–	–	*χ*^2^ = 27.0**
Postnatal diagnosis (*N*)	61 (55%)	NA	NA	7 (26%)	20 (49%)	14 (64%)	20 (100%)	–	–	
SES^a (MacArthur Score ^b) M (SD)	46.9 (18.9)	39.9 (17.2)	46.8 (18.6)	43.2 (16.9)	52.9 (18.9)	58.2 (19.4)	47.9 (19.3)	*t* = − 2.9*	*F* = 4.5**	*F* = 3.0*

The MacArthur Scale of Subjective Social Status. Psychosocial Research Notebook

*p < .05

**p < .001

^a Socioeconomic status

^b Adler, N. E., Stewart, J., et al. [46]

### Measures

For the SCA groups, intellectual functioning was estimated using Full Scale IQ (henceforth “IQ”) from the Wechsler Intelligence Scale for Children, Fifth Edition (WISC-V [47];) or the Wechsler Adult Intelligence Scale, Fourth Edition (WAIS-IV [48];). For the typically developing groups, IQ was measured using the Wechsler Abbreviated Scale Intelligence, Second Edition (WASI-II, [49]). IQ ratings are expressed as a standard score (*M* = 100; SD = 15) in comparison to normative expectations based on age. Higher scores indicate higher intellectual functioning.

Psychopathology for all participants was measured using Child Behavior Checklist (*N* = 219; CBCL [50];) and Adult Behavior Checklist (*N* = 22; ABCL [51];) completed by each participant’s caregiver who knew the participant well (e.g., parent). For simplicity, scales from both measures will henceforth be referred to as CBCL subscales, as data were combined across the CBCL and ABCL, and most participants received the CBCL. This widely used instrument combines item-level responses to derive eight syndrome scales (anxious/depressed, withdrawn/depressed, somatic complaints, social problems, thought problems, attention problems, rule-breaking behavior, and aggressive behavior), and three broadband scales: externalizing domain (rule-breaking behavior, aggressive behavior), internalizing domain (anxious/depressed, withdrawn/depressed, somatic complaints), and total problems (items across all syndrome scales plus an additional 17 items that do not belong to any syndrome scale). Ratings on each scale are expressed as *T*-scores (*M* = 50; SD = 10) in comparison to normative expectations based on age and gender. Higher scores indicate the presence of more problem behaviors. For the eight syndrome scales, *T*-scores of 65–69 are borderline clinical, and *T*-scores of 70 or higher are in the clinical range. For the three broadband scales T-scores of 60–63 are borderline clinical and T-scores of 64 or higher are clinically elevated.

### Statistical analysis

We examined the omnibus effect of karyotype group on each of the 11 CBCL-derived dimensions of psychopathology using ANOVAs—once considering all groups, and once restricted to just the four SCA groups (applying Bonferroni correction each time). We also used *t* tests to (i) compare the distribution of CBCL scores in SCA participants (all karyotypes combined) as compared to euploidic controls within our sample (2 sample *t* tests with Bonferroni correction) and (ii) compare karyotype-specific score distributions for each CBCL scale against the reference norm mean *t*-score of 50 (one sample t-tests with Bonferroni correction). To determine the degree to which any SCA karyotype group effects on CBCL scores existed above and beyond co-occurring variation on general cognitive ability, we also tested for omnibus effects of SCA group on each CBCL dimension while covarying for IQ.

**Fig. 1:**
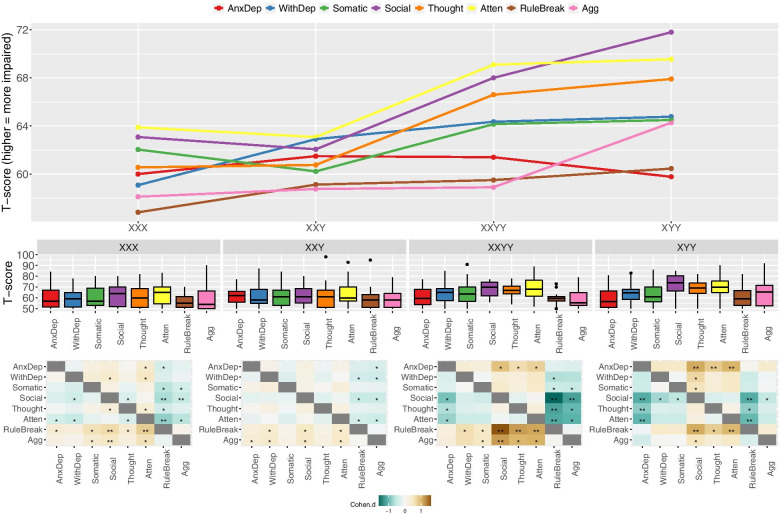
Tripartite visualization comparing CBCL scores across and within SCA groups. **A** Line plot showing profiles of CBCL subscale scores across different SCA groups. **B** Boxplots showing distributions of different CBCL subscale scores within each SCA group. **C** Heatmaps showing pairwise CBCL subscale comparisons within each SCA. Colors denote effect sizes, and asterisks denote statistical significance (*nominal *p* < 0.05, **Bonferroni-corrected *p* < 0.05)

**Fig. 2:**
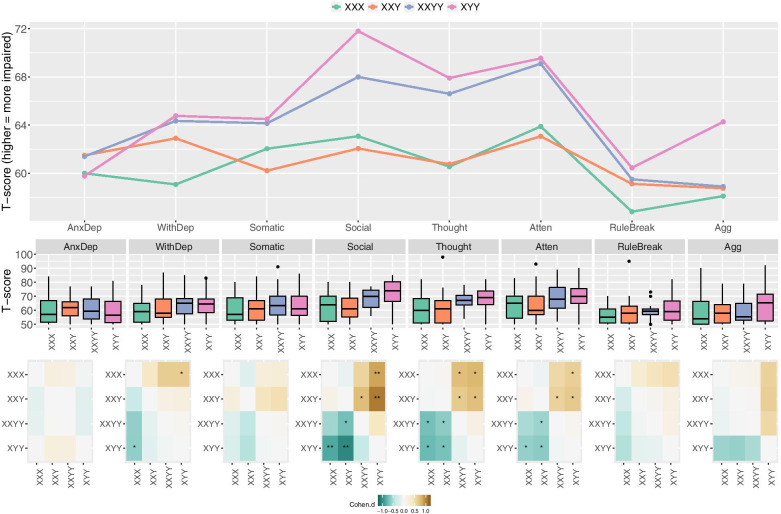
Tripartite visualization comparing SCA groups across CBCL subscales. **A** Line plot showing score profiles for each SCA group across different CBCL subscales. **B** Boxplots showing score distributions for different SCA groups within each CBCL subscale. **C** Heatmaps showing pairwise SCA group comparisons within each CBCL subscale. Colors denote effect sizes, and asterisks denote statistical significance (*nominal *p* < 0.05, **Bonferroni-corrected *p* < 0.05)

Variation in CBCL subscale scores across SCA groups were visualized using tripartite plots comprising: (i) dot-line plots showing profiles of CBCL subscale scores across SCA groups (Fig. 1A), and profile of SCA group scores across the eight CBCL syndrome subscales (Fig. 2A), (ii) boxplots showing the distribution of scores per CBCL subscale for each SCA group (Fig. 1B), and scores per SCA group for each CBCL subscale (Fig. 2B), and (iii) heatmaps showing Cohen’s *d* and *p* values for pairwise t-test comparisons between CBCL subscales per SCA group (Fig. 1C), and between SCA groups per CBCL syndrome subscale (Fig. 2C). The statistical tests represented by these heatmaps provide a fine-grained analysis of patterned SCA karyotype effects on different dimensions of psychopathology. These comprehensive pairwise t-tests were planned *a priori* (i.e., not post hoc to omnibus tests) and Bonferroni corrected for multiple comparisons [Fig. 1C: between scales per karyotype, *p* = (0.05/28); Fig. 2C: between karyotypes per scale, *p* = (0.05/6)].

**Fig. 3:**
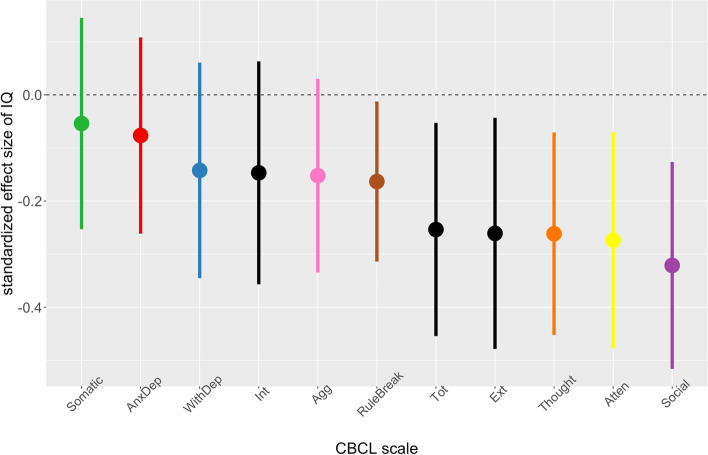
Associations with IQ for different domains of psychopathology in SCA. For each CBCL scale (*x*-axis), we provide the estimated standardized regression coefficient for IQ (*y*-axis: point = estimated coefficient, line = 95% confidence intervals of coefficient), which estimates the standard deviation shift in CBCL scores for 1 standard deviation increases in IQ

Finally, to examine potential relationships between psychopathology and cognitive ability in SCA we first used general linear models to predict variation in each of the 11 CBCL subscale scores as a function of IQ, SCA karyotype and the interaction between these two terms. The omnibus *F*-test associated with this interaction term tests the hypothesis that psychopathology-IQ associations vary between SCA groups. In the absence of a significant interaction term, the beta coefficient for the main effect of IQ estimates the relationship between IQ and CBCL subscale scores, controlling for the main effect of the SCA group (Table 2, Fig. 3). To provide a full visualization of all psychopathology-IQ relationships represented in our dataset we generated a matrix of scatterplots between IQ and CBCL scores, faceted by CBCL subscale and SCA karyotype group (Fig. S1). Each pairwise relationship was quantified using the percentage bend robust regression coefficient.

**Table 2:** Variables of interest

								Statistic			
	Total	XX	XY	XXX	XXY	XYY	XXYY	SCA vs. Controls (t-test)	Comparing all groups (ANOVA)	Comparing SCA groups only (ANOVA)	Comparing SCA groups covarying for IQ (ANCOVA)
**IQ standard scores** ***M*** **(SD)**											
Full-scale IQ	106.0 (16.4)	113.5 (11.9)	116.2 (10.3)	95.4 (15.3)	101.6 (13.8)	90.6 (14.9)	85.1 (12.3)	*t* = 11.3**	*F* = 35.5**	*F* = 6.8**	–
**CBCL**^**e**^**/ABCL**^f^ ***T*-scores** ***M*** **(SD)**											
Total problems	50.9 (14.8)	39.5 (8.0)	41.1 (8.8)	59.7 (12.3)	61.5 (10.6)	68.0 (8.1)	66.5 (6.9)	*t* = − 18.2**	*F* = 76.5**	*F* = 3.8*	*F* = 3.6*
Internalizing	51.7 (13.4)	42.4 (7.4)	43.1 (8.8)	60.3 (11.3)	62.0 (10.8)	63.7 (9.3)	64.8 (8.9)	*t* = − 16.2**	*F* = 55.4**	*F* = 0.9	*F* = 0.9
Externalizing	49.4 (11.9)	42.1 (7.6)	43.1 (7.2)	53.5 (13.7)	56.9 (10.7)	62.5 (10.2)	58.8 (8.1)	*t* = − 11.9**	*F* = 33.8**	*F* = 2.8*	*F* = 2.7
Anxiety/depression	55.6 (7.9)	50.9 (1.9)	51.5 (2.8)	60.0 (9.8)	61.5 (8.2)	59.8 (9.5)	61.4 (9.3)	*t* = − 10.9**	*F* = 27.5**	*F* = 0.3	*F* = 0.3
Withdrawn	56.4 (9.2)	51.0 (2.7)	51.4 (4.0)	59.1 (8.4)	62.9 (10.8)	64.8 (10.5)	64.4 (9.2)	*t* = − 11.4**	*F* = 32.4**	*F* = 1.7	*F* = 1.5
Somatic	56.1 (8.8)	51.8 (2.8)	52.3 (4.4)	62.0 (9.7)	60.2 (8.4)	64.5 (10.8)	64.2 (11.0)	*t* = − 10.3**	*F* = 25.9**	*F* = 1.2	*F* = 1.3
Social	57.2 (9.9)	50.3 (1.2)	51.8 (4.0)	63.1 (9.8)	62.1 (8.7)	71.8 (10.7)	68.0 (7.4)	*t* = − 13.4**	*F* = 59.0**	*F* = 5.6*	*F* = 3.6***
Thought	56.7 (9.3)	50.8 (2.3)	51.5 (3.2)	60.6 (9.7)	60.8 (10.4)	67.9 (10.0)	66.6 (6.6)	*t* = − 12.2**	*F* = 43.0**	*F* = 4.2*	*F* = 3.8*
Attention	57.7 (10.3)	51.0 (2.1)	51.0 (2.1)	63.9 (9.7)	63.1 (10.7)	69.6 (9.4)	69.1 (10.6)	*t* = − 14.5**	*F* = 56.2**	*F* = 3.0*	*F* = 2.5
Rule breaking	54.7 (6.8)	51.2 (2.4)	51.1 (2.5)	56.8 (7.1)	59.1 (8.7)	60.5 (8.4)	59.5 (5.3)	*t* = − 10.1**	*F* = 25.0**	*F* = 1	*F* = 0.9
Aggressive	54.9 (8.0)	50.8 (2.2)	50.9 (2.1)	58.1 (10.0)	58.8 (7.7)	64.3 (12.0)	58.9 (8.4)	*t* = − 9.6**	*F* = 25.3**	*F* = 2.2	*F* = 1.7

*Uncorrected *p* < .05

**uncorrected *p* < .001

***Bonferroni corrected *p* < .05

^e^Childhood Behavior Checklist

^f^Adult Behavior Checklist

Statistical analyses were performed using R software [52]), and SPSS Statistics Versions 25 [53] and 26 [54]. The following R packages were used in data analysis and visualization: ggplot2 [55], and patchwork [56].

## Results

### Profiling SCA effects on psychopathology

All CBCL subscale scores showed statistically significant differences between the euploidic and aneuploidic groups in two-sample *t*-tests (all |T| > 9, and *p* < 0.001), as well as statistically significant omnibus effect of karyotype in one-way ANOVAs across XX, XY, XXY XYY, XXX, and XXYY groups (all *F* > 25 and all *p* < .001, Table 2). We also observed that all subscale scores in all SCA groups—with the notable exception of the externalizing problems broadband scale in XXX syndrome—were significantly elevated above reference norms (one-sample *t*-test against a *T*-score of 50) after Bonferroni correction for multiple comparisons (all corrected *p*’s < .05).

One-way ANOVAs comparing the four SCA groups alone indicated a statistically significant effect of karyotype group at the uncorrected *p* < .05 level on the following scales: total problems (*F*(3,109) = 3.8), externalizing *F*(3,109) = 2.8), social *F*(3,109) = 5.6), thought *F*(3,109) = 4.2), and attention problems *F*(3,109) = 3.0) (Table 2). The total problems, and social and thought problems scales survived Bonferroni correction for multiple comparisons across CBCL scales (i.e., *p* < .05/11). These data support the notion that different SCA karyotypes are associated with partly distinct profiles of psychopathology. The profile of CBCL scores across SCA groups and the profile of SCA group scores across CBCL scales are shown in Figs. 1 and 2A and A, respectively. Accompanying boxplots show CBCL score distributions by scale within the SCA group (Fig. 1B) and by the SCA group within scale (Fig. 2B).

Pairwise *t* tests comparing CBCL subscales within SCA karyotype groups (Fig. 1C), and SCA karyotype groups within each CBCL subscale (Fig. 2C) identified the salient contrasts driving variation in CBCL scores across SCA groups (Table 2). Comparison of CBCL subscales within each SCA group (Fig. 2B, C) revealed a relatively even elevation of all subscales in XXY syndrome, while the other three groups each featured specific subscales with notably high or low score elevations. Specifically; the XYY group showed significant elevation of social, thought and attentional problems relative to anxious-depressed and rule breaking problems; the XXYY group showed significant elevation of social, thought and attentional problems relative rule breaking problems, and significant elevation of social problems relative to aggressive behaviors; and the XXX group showed significant elevation of social, and attentional problems relative to rule breaking, and of social problems relative to aggressive behavior (Fig. 1C). Complementing these within-group analyses, comparisons between SCA groups for each CBCL scale indicated that social, thought and attentional problems tended to be more elevated in XYY and XXYY groups as compared to XXY and XX groups (significantly so after Bonferroni correction for social problems). A corollary of the more pronounced impact of XYY and XXYY karyotypes on these domains of psychopathology is that these groups showed the greatest dispersion in mean scores across the measured domains of psychopathology (Table 2, Fig. 1A). Specifically, the CBCL *T*-score standardized deviations (i.e., CBCL score increments of 10) between the highest and lower subscale score in each SCA subgroup were: 1.2 for XYY, 1 for XXYY, 0.7 for XXX, and 0.4 for XXY.

Taken together, the results above indicate that (i) all four of the SCA groups studied are associated with a broad increase in risk for psychopathology, but that social and attentional difficulties tend to be the most strongly impacted subdomains, and (ii) the apparent selective vulnerability of social and attentional domains relative to other aspects of psychopathology is particularly pronounced in SCA groups bearing an additional Y chromosome (XYY and XXYY).

### Coupling between IQ and psychopathology in SCAs

We did not find evidence of statistically significant modulation of IQ-psychopathology relationships by SCA karyotype group (uncorrected *p*-value range: 0.16-0.78 across CBCL subscales). However, given the rarity of this cross-SCA dataset (and associated limitations in statistical power to detect such higher-order interactions), we generated an exploratory visualization of psychopathology-IQ relationships for each unique CBCL subscale-SCA group combination (Fig. S1). Qualitatively, this revealed a wide range in estimated correlations between IQ and psychopathology. Specifically, some psychopathology correlations with IQ were moderately negative [e.g., with thought problems in XXY (*r* = − 0.48), social problems in XYY (*r* = − 0.47), and social problems in XXX (*r* = − 0.43)], while others were near zero (e.g., rule breaking in XXYY, somatic problems in XXX, and thought problems in XYY).

Given the absence of statistically significant modulation of psychopathology-IQ relationships by SCA group (IQ*group interaction Bonferroni-corrected *p* values > 0.05 for all CBCL subscales), we estimated the standardized regression coefficient for IQ on each CBCL subscale controlling for the main effects of SCA group (Fig. 3). These analyses revealed that across all SCA groups, associations with IQ were strongest for social, attentional, and thought problems (standard deviation [SD] shifts up in psychopathology for 15-point drop in IQ: 0.32, 0.27, and 0.26 respectively), and weakest for domains of somatic problems and anxious-depressed domains (SD shifts up in psychopathology for 15-point drop in IQ both < 0.08). While controlling for IQ, SCA group showed a statistically significant omnibus *F*-test for social problems after correction for multiple comparisons across CBCL scales.

## Discussion

The current study adds to our understanding of psychopathology in SCA in a number of key directions as detailed below.

### Broad risk for psychopathology across SCAs

Our findings in this previously unpublished dataset of behavioral measures in multiple SCA groups add to the mounting consensus that carriage of extra X- and or Y-chromosomes in humans increases risk for diverse domains of psychopathology [1–3]. Specifically, we observe that almost all domains of psychopathology across all SCA groups were significantly elevated relative to reference norms and study specific controls, suggesting that carriage of extra sex chromosomes results in a broad increase in risk for psychological and behavioral problems across multiple domains. Increases in psychopathology associated with SCA are greatest for measures of total psychopathology, and problems in attentional and social subdomains. These findings reinforce prior reports to underscore the need for standard care in SCAs to be expanded to routinely include psychological screening and referral for treatment as indicated.

### Evidence for differential psychopathological risk as a function of SCA karyotype

We provide new evidence for variation in psychopathology across different domains of measurement and different SCAs. Specifically, our findings point to the clinically important observation that social problems are not only one of the domains of psychopathology that are most impacted by SCAs in general (see above) but is also the domain that varies most between different SCA groups. At a relaxed level of statistical significance (i.e., uncorrected *p* < .05), we also find some evidence for variation in levels of total psychopathology and externalizing, somatic, attention and thought problems across SCA subgroups. Of note, however, findings for the CBCL thought problems scale should be interpreted with caution as this scale is not necessarily indicative of a thought disorder but also consists of items measuring obsessions, repetitive behaviors, and sleep disturbance [57].

We find that the statistically significant variation in social problems (and to a nominally significant extent, total psychopathology, externalizing, and attentional problems) across SCA groups is driven by higher elevations in groups carrying an extra Y- as compared to an extra X-chromosome. Thus, carriage of an extra Y-chromosome appears to have a particularly pronounced impact on social and attention problems, as well as problems associated with the CBCL thought problems subscale. These findings are generally consistent with the few existing studies that have compared measures of psychopathology across SCA groups. For example, SCAs defined by carriage of an extra Y-chromosome have shown higher rates of autism spectrum disorder (ASD) and ADHD diagnoses [10, 20, 58], as well as higher scores on continuous measures of attentional problems [10], as compared to SCAs defined by carriage of an extra X-chromosome. Of note, our data do not support the notion that problems with externalizing and aggressive behaviors are more pronounced in SCAs defined by supernumerary Y- vs. supernumerary X-chromosomes [1, 3, 4, 59]. This lack of statistically significant group differences is associated with weak effect sizes. We therefore interpret this observation as providing further evidence against the previously refuted (yet still actively stigmatizing) notion that carriage of additional Y chromosomes is preferentially associated with aggressive and anti-social behaviors [1]. We also fail to replicate prior reports that carriage of an extra X chromosome has a greater impact on internalizing symptoms than carriage of an extra Y [3]. Further research in larger cohorts using standardized measures will likely help to resolve these inconsistencies and more definitively resolve the potentially patterned effects of X- and Y-chromosome aneuploidies on different domains of psychopathology. Nevertheless, we provide evidence that different SCAs may impart slightly different profiles of risk, and that clinical assessment should be attuned to these potential differences.

The potential biological bases for differential Y- vs. X-chromosome effects on social functioning remain unclear. It is notable that disorders of social functioning like ASD and psychopathology are more common in XY males as compared to XX females in the general population [60], raising the idea that the Y-chromosome may have a special influence on brain systems subserving social functioning. However, although there is evidence for anatomical changes in social networks within the human brain with Y-chromosome aneuploidy, these same systems are also sensitive to X-chromosome dosage [30, 61, 62] - which suggests that traditional in vivo measures of regional brain anatomy may not prove useful in identifying markers of the differential psychopathological risks imparted by different SCA karyotypes. It is well established however that the proximal effects of X- and Y-chromosome aneuploidy on human gene expression are highly divergent [63] (e.g., X- but not Y-dosage effects on genes escaping X-inactivation)—so, there is a pressing need to understand if and how these divergent effects on gene expression might manifest in brain development as a potential source for downstream clinical variation among SCA groups.

### Relationship between psychopathology and intellectual functioning in SCA

To our knowledge, the current study provides the first comparisons of IQ and psychopathology across SCA genotypes and domains of psychopathology. We do not find evidence for statistically significant modulation of IQ-psychopathology relationships as a function of SCA karyotype. However, our observation of varying correlation coefficients (spanning the range 0–|0.48|) in modestly sized dataset hints that different domains of psychopathology may vary in their relationships with IQ as a function of SCA karyotype (Fig. S1). However, there is growing recognition that correlations between different dimensions of cognition and psychopathology can be weak at the population level and require large sample sizes to be reliably estimated [64], which poses a particular challenge for estimating such correlations within individually rare SCA subgroups. Nevertheless, it is notable that some of the relationships between IQ and psychopathology that have been reported in other datasets (autism spectrum disorder [65];, and anxiety [66];)—were not apparent in SCA. Thus, there remains an open question as to whether relationships between IQ and psychopathology show substantial variation between different clinical subgroups.

Across our full SCA cohort, when controlling for the main effect of the SCA group, we find that different domains of psychopathology can vary in the strength of their relationship with IQ, with social and attentional problems being most strongly coupled to IQ variation. This observation is important from a clinical perspective as it recommends particularly close assessment of cognition in individuals with SCA showing pronounced social and/or attentional problems, and vice versa. From a biological perspective, the close coupling between socio-attentional and general cognitive deficits suggests that these two domains of functioning may share closely overlapping brain substrates. This notion is supported by emerging evidence that general cognitive functioning is closely tied to functional organization of specific cortical networks (default mode and dorsal attentional) that are also key components of social and attentional systems within the brain [67].

## Limitations and future directions

Our findings should be considered in light of several caveats and limitations. First, it is well recognized that a considerable proportion of individuals carrying extra sex chromosomes remain undetected. As such studies of clinically identified cohorts such as ours may provide inflated estimates of penetrance due to ascertainment bias [3, 68, 69]. Such inflation is likely to be less for SCA subgroups with more severe presentations (i.e., XXYY vs. XXY for example [19])—providing a potential methodological source for differing presentations between clinically ascertained groups. However, we detect substantial differences in psychopathology between SCA subgroups with similar rates of under-detection (e.g. XXX and XXY [69])—suggesting that karyotype-specific ascertainment biases cannot be the sole driver of our observed subgroup differences. Expansion of SCA cohort sizes in future research will enable adequately powered statistical comparisons of carriers diagnosed prenatally vs those diagnosed postnatally, which can help to provide a proxy for potential ascertainment biases [70].

Larger cohorts will also facilitate characterization of rare SCA tetra- and pentasomy groups (e.g., XXYY and XXXXY respectively). Indeed, the future study of larger, and more karyotypically diverse SCA cohorts will enable statistical modeling of ordinal X- and Y-chromosome dosage effects on behavioral functioning/psychopathology, intellectual functioning, and relationships between the two, as opposed to categorical group-based comparisons. Such an approach could also include a more mechanistic examination of the factors that mediate the path between genotype and behavioral phenotype through studying variability in intermediate biological phenotypes (e.g., neuroimaging-derived) across karyotypes as potential mediators of discrepant profiles of psychopathology in SCA. Second, our dataset is cross-sectional in nature, and the low population prevalence of SCAs complicates the collection of the large longitudinal datasets that would be required to model age-varying psychopathology. An important goal for future work will be to better understand how the impact of SCA on mental health may vary across the lifespan. Third, although our study considers multiple domains of psychopathology across multiple SCA groups, there is a pressing need for even more multidimensional measurement of clinical features in SCA, including the many continuous (e.g., temperament) and categorical (e.g., psychiatric diagnoses) variables that were not included in our report. Identifying how variability in these dimensions can impact responsiveness to social/behavioral interventions, at the individual and group level, is also an important undertaking for future research.

## Conclusion

Notwithstanding the above caveats, our study builds on prior work to provide a more detailed understanding of the patterning of psychopathology across different SCA groups, and how variation in psychopathology within SCAs related to co-occurring variation in general cognitive ability. Our findings may help to better target clinical assessments of affected individuals and inform thinking about potential biological factors that might organize the patterning of neuropsychiatric difficulties in X- and Y-chromosome aneuploidies.

## Supplementary Information

**Additional file 1: Figure S1.** Exploratory Visualization of Psychopathology-IQ Relationships by SCA Group and CBCL scale. Each point is a person, and IQ-CBCL score associations are shown for each unique SCA group-CBCL scale combination. Fit lines are from general linear models, and provided correlation coefficients are percentage bend robust regression coefficients.

**Additional file 2: Supplementary Table 1.** Demographic variables.

## Data Availability

The datasets generated and/or analyzed during the current study are not publicly available to protect individual patient privacy. Data are available upon reasonable request.

## Abbreviations

SCA: Sex chromosome aneuploidy
ADHD: Attention deficit/hyperactivity disorder
KS: Klinefelter syndrome
IQ: Intelligence quotient
TD: Typically developing
NIH: National Institutes of Health
WISC: Wechsler Intelligence Scale for Children
WAIS: Wechsler Adult Intelligence Scale
WASI: Wechsler Abbreviated Scale of Intelligence
CBCL: Childhood Behavior Checklist
ABCL: Adult Behavior Checklist
ANOVA: Analysis of variance
ASD: Autism spectrum disorder
SD: Standard deviation
