# Supplementary figures and images for: Patterns of psychopathology and cognition in sex chromosome aneuploidy
Source: J Neurodev Disord. 2021 Dec 15;13:61. doi: 10.1186/s11689-021-09407-9 (PMC8903493; doi:10.1186/s11689-021-09407-9)

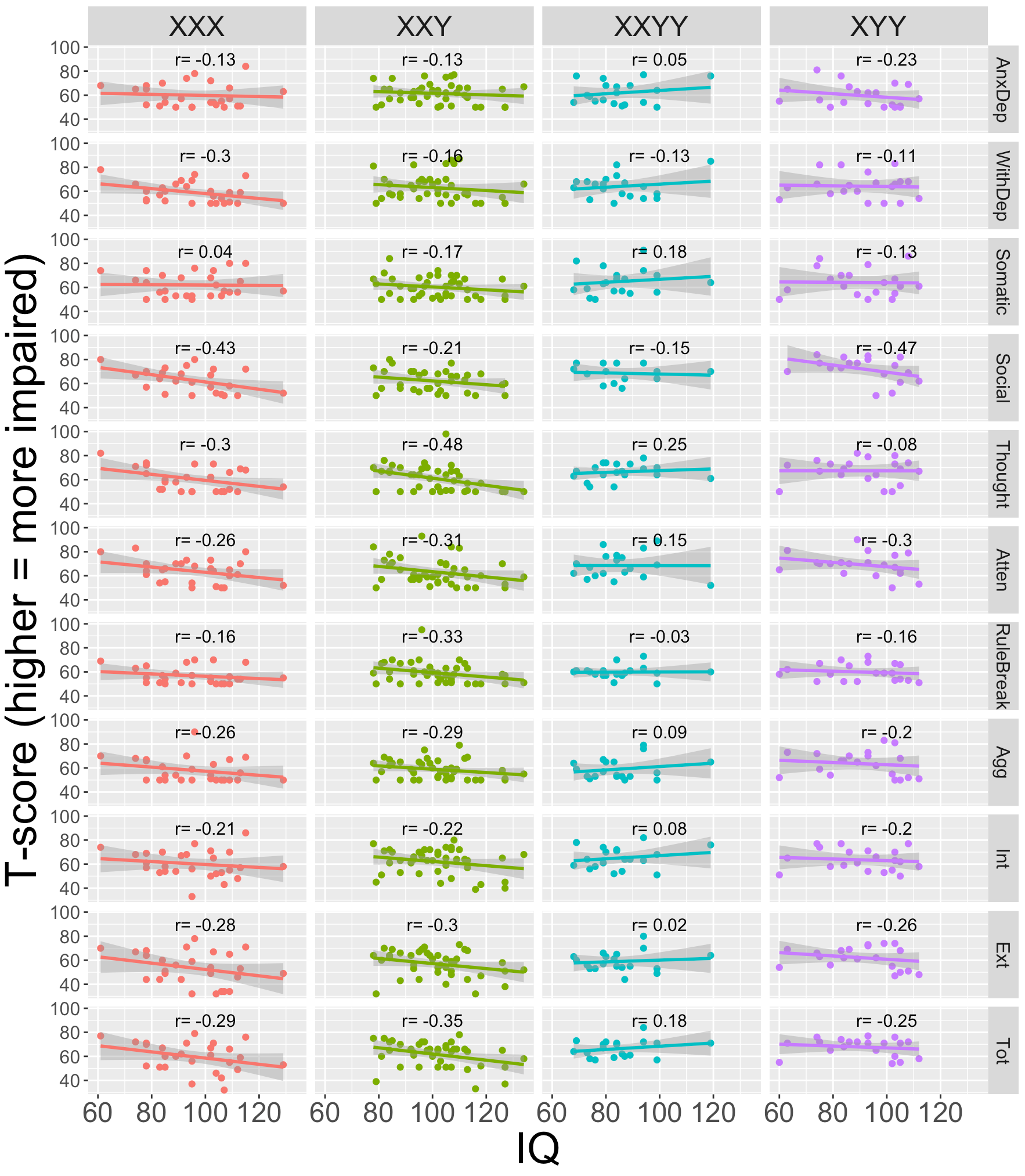

Supplement: Supplementary file 1 — Additional file 1: Figure S1. Exploratory Visualization of Psychopathology-IQ Relationships by SCA Group and CBCL scale. Each point is a person, and IQ-CBCL score associations are shown for each unique SCA group-CBCL scale combination. Fit lines are from general linear models, and provided correlation coefficients are percentage bend robust regression coefficients. [file 11689_2021_9407_MOESM1_ESM.tiff]
